# ACCULTURATION ON PSYCHOLOGICAL DISTRESS AMONG OLDER KOREAN AMERICANS: DOES ETHNIC COMMUNITY SOCIAL CAPITAL MATTER?

**DOI:** 10.1093/geroni/igac059.1909

**Published:** 2022-12-20

**Authors:** Juyoung Park, Maria Aranda, Yeon Jin Choi, Yuri Jang

**Affiliations:** University of Southern California, Los Angeles, California, United States; University of Southern California, Los Angeles, California, United States; University of Southern California, Los Angeles, California, United States; University of Southern California, Los Angeles, California, United States

## Abstract

Responding to the rapid growth of the older immigrant population and building upon the literature on the critical role of acculturation in older immigrants’ health and well-being, we focused on the role of ethnic community social capital (social cohesion, social engagement, safety, and negative interactions in ethnic communities) in older Korean Americans. Guided by social capital and stress-buffering theories, we examined the direct effect of acculturation and ethnic community social capital on psychological distress, as well as their interactions. We hypothesized that the negative impact of low acculturation on mental health would be lowered by positive perceptions of and experiences in ethnic communities. Using data from 2,150 participants in the Study of Older Korean Americans (Age range = 60-99, M [SD] = 73.4 [7.97]), the direct and interactive effect models were examined. Results showed that low acculturation posed a significant risk to mental health and all four types of ethnic community social capital had a significant direct effect. Furthermore, significance was observed in the interaction of acculturation with social cohesion (B [SE] = .01 [.01]. p < .05) and with negative interaction (B [SE] = -.01 [.01]. p < .01). The negative impact of low acculturation was attenuated among those with a high sense of ethnic community social cohesion but intensified among those with frequent experiences of negative interactions with ethnic community members. Our findings highlight the importance of social capital that forms within ethnic communities and provide implications for programs and services to promote older immigrants’ mental well-being.

